# Novel antimicrobial peptides and peptide-microbiome crosstalk in Appalachian salamander skin

**DOI:** 10.1038/s41522-025-00837-0

**Published:** 2025-11-20

**Authors:** Carly R. Muletz-Wolz, Julian Urrutia-Carter, Owen Osborne, Steve Kutos, Jose Meneses Montano, Joseph D. Madison, Brian Gratwicke, Ratanachat Racharaks, Norma E. Roncal, Randall R. Jimenez, Amy Ellison, Timothy P. Cleland

**Affiliations:** 1 Center for Conservation Genomics, Smithsonian’s National Zoo & Conservation Biology Institute, Washington, DC USA; 2 Department of Biological Sciences, Bangor University, Bangor, UK; 3 Department of Biology, Xavier University of Louisiana, New Orleans, LA USA; 4 Center for Species Survival, Smithsonian’s National Zoo & Conservation Biology Institute, Front Royal, VA USA; 5 Walter Reed Army Institute of Research, Bethesda, MD USA; 6 International Union for Conservation of Nature, San Jose, Costa Rica; 7 Smithsonian Museum Conservation Institute, Suitland, MD USA; 8 Present Address: Complex Microbial Systems Group, National Institute of Standards & Technology, Gaithersburg, MD USA

**Keywords:** Applied microbiology, Microbial communities, Environmental microbiology, Pathogens

## Abstract

Using multi-omics tools, we discovered new antimicrobial peptides (AMPs) and examined AMP-microbial interactions in three Appalachian salamander species (*Plethodon cinereus*, *Eurycea bislineata* and *Notophthalmus viridescens*). We conducted skin transcriptomics (*n* = 13) and proteomics (*n* = 91) to identify 200+ candidate AMPs. With candidate AMPs, we identified correlations with skin microbiomes and synthesized 20 peptides to challenge against pathogens of amphibians (*Batrachochytrium dendrobatidis: Bd*) and humans (ESKAPEE). Using transcriptomics, candidate AMPs were detected in all individuals with Cathelidicins being most common. Using proteomics, AMPs were found in 34% of individuals (31/91)—predominately *E. bislineata*—with Kinin-like peptides being most common. Candidate AMP composition generally predicted skin bacterial composition, suggesting that AMPs influence host-microbial symbioses. Crude and synthesized peptides showed limited activity against Bd. Two synthesized Cathelicidins (Pcin-CATH3 and Pcin-CATH5) inhibited human pathogens, *Acinetobacter baumannii, Pseudomonas aeruginosa* and *Escherichia coli*. Our findings inform the potential usage of AMPs in conservation and translational applications.

## Introduction

Animal immune systems establish intimate relationships with host microbiota. Antimicrobial peptides (AMPs) or “host-defense peptides” are major players in the microbial-immune interface in multicellular organisms, in which they can kill unwanted microbes, while presumably allowing mutualistic or commensal microbes to persist. Most animal species express multiple distinct AMP genes in epithelial tissues and in response to pathogens^1^. However, only a few studies have examined the relationship between AMPs and resident host microbiomes^2–5^, and to our knowledge no comparative study exists using vertebrate taxa to examine AMP-microbiome interactions. AMPs are a promising alternative to traditional antibiotics and offer the potential for applications in disease mitigation in conservation^6^ and in human medicine^7^. Most AMPs discovered to date originate from frogs (1000+), arthropods (500+) and mammals (300+)^1,8^. To harness the full potential of AMPs in translational applications, mining all domains of life and using new -omics and mathematical tools will fuel their discovery and use. Here, we focus on discovering AMPs and their natural biology in Appalachian salamanders. To our knowledge, a total of nine AMPs have been described in salamanders: Aurein-like AMP Ramosin^9^, lipopeptide Antillatoxin B^10^, β-defensin CFBD^11^, Cathelicidin AdCath^12^, Andricin B^13^, and four uncharacterized AMPs^14^.

Amphibian skin is a biologically rich environment. AMPs are produced and secreted from granular glands in amphibian skin and show broad-spectrum antimicrobial activity and immunomodulatory properties^8,15,16^. The skin of amphibians is also inhabited by a diverse community of bacteria, referred to as the microbiome, that co-exist with host AMPs. Amphibians’ first lines of defense against pathogens are AMPs and the skin microbiome, which are considered part of the amphibian innate immune system. Frogs have been the primary focus of AMP research for decades, while AMP diversity in salamanders has been poorly studied^17^. Of the nine salamander AMPs discovered to date, some show structural similarities to known families of frog AMPs^9,11,12^, while others show levels of structural uniqueness that may warrant definition of new families of AMPs^13,14^. It is important to note that while salamander AMPs show structural similarities to known frog AMPs, their sequence diversity is unique from that of frogs^17^. Insight into the AMP-microbial interface is critical to understanding disease outcomes from pathogens, which are increasingly devastating to both frogs and salamanders^18,19^, but also applies broadly across plants and animals that are also being negatively impacted by emerging infectious diseases^20–22^. Furthermore, AMPs and microbiomes may facilitate rapid host adaptation to environmental change, such as disease^23^ or climate change^24,25^, as they have the potential to change and respond rapidly to their environment^26–28^.

From a conservation perspective, infectious diseases have played a significant role in global amphibian declines. Particularly, the disease chytridiomycosis caused by skin infection by either of two fungal species, *Batrachochytrium dendrobatidis* (Bd) or *Batrachochytrium salamandrivorans* (Bsal). Bd occurs globally infecting over 500 species of frogs and salamanders, and has been linked to declines in the United States, Central and South America, Europe and Australia^18^. Bsal has recently emerged with incidences of declines observed in Europe^19^; major concerns exist about further global dispersal of Bsal into biodiversity hotspots, such as Appalachian salamanders^29^. The presence of specific peptides or bacterial species^30^ can reduce amphibian susceptibility to Bd infection and can even work synergistically to kill Bd^31^. Identifying the molecular and/or microbial mechanism of pathogen protection is critical to informing conservation strategies such as targeted breeding (e.g., selecting for defensive AMPs in captive populations prior to release) and probiotic therapy (e.g., selecting for defensive microbiomes in susceptible wild populations). AMPs likely play an important role in structuring the amphibian skin microbiome^32^ and interact with probiotics during inoculation^16^. Understanding AMP-microbial interaction will improve effectiveness of conservation strategies in disease mitigation.

From a human medicine perspective, AMPs are an attractive candidate for translational application with several in clinical trials^7^. The mechanism of AMP action is to integrate into the microbial cell membrane and disrupt its integrity, resulting in cell lysis. Many AMPs show broad-spectrum killing ability in which those that kill fungi (such as Bd) can also kill bacteria and viruses^1,33,34^. AMPs may offer an alternative to antibiotics used to treat multi-drug resistant (MDR) bacterial infections as they are biochemically simple (15 – 50 amino acids) and have highly efficient killing mechanisms, which are predicted to prevent bacterial resistance.

Here, we integrate transcriptomics, proteomics and microbial ecology to achieve three objectives focused on AMP discovery and AMP-microbial interactions in three Appalachian salamander species (*Eurycea bislineata*, *Plethodon cinereus* and *Notophthalmus viridescens*). The first objective was to compare how different -omics methods impact identifying candidate AMPs (i.e., peptides that show homology to known antimicrobial peptides). Specifically, we compared skin peptide profiles discovered using (i) skin gene expression via transcriptomics and (ii) peptide presence via proteomics with peptide release elicited with one of two methods: acetylcholine injection or massage. Our second objective was to examine the relationship between peptide diversity and microbial diversity on salamander skin. Based on studies in frog species, we anticipated identifying a high diversity of AMPs (~5–45 peptides per species) across the three salamander species with little overlap between species^6,35,36^, and expected that peptides act as a selective pressure driving skin microbiome assembly^32,37^. Our third objective was to characterize antimicrobial activity of crude peptides and synthesized peptides against Bd and MDR bacteria. Together, AMPs and host microbiomes are the first lines of defense against pathogen infection and understanding their relationship is interesting from both a basic and applied scientific standpoint.

## Results

Bd was detected on all species, with low Bd loads and prevalence in *E. bislineata* and *P. cinereus*, contrasting sharply to high Bd loads and prevalence in *N. viridescens* (Table 1). Bsal or ranavirus were not detected.

**Table 1:** Sample sizes and Bd prevalence per species per site within three localities: Mountain Maryland (MD), the Smithsonian National Zoo & Conservation Biology Institute (NZCBI) campus in Front Royal, VA (Front Royal CBI) and George Washington Jefferson National Forest (GWJNF) in Virginia

Species	Locality	Site code	Bd prevalence	Sample size acetylcholine	Sample size massage	Sample size transcriptomics
*E. bislineata*	Front Royal CBI	POSH	38%	8	--	--
*E. bislineata*	GWJNF	HOQ	20%	3	2*	2*
*E. bislineata*	GWJNF	TIH	0	4	--	--
*E. bislineata*	Mountain MD	DMP	0	5	--	--
*E. bislineata*	Mountain MD	SRBR	0	3	--	--
*P. cinereus*	Front Royal CBI	POSH	0	8	--	2
*P. cinereus*	GWJNF	FLAK	20%	4	--	1
*P. cinereus*	GWJNF	TIMR	0	3	--	--
*P. cinereus*	Mountain MD	SRBE	0	4	--	2
*P. cinereus*	Mountain MD	DMP	0	6	6	--
*N. viridescens*	Front Royal CBI	LEEP	18%	9	--	2
*N. viridescens*	GWJNF	GAR	92%	5	7 (2*)	2*
*N. viridescens*	GWJNF	WOF	100%	4	--	--
*N. viridescens*	Mountain MD	DMP	80%	5	--	--
*N. viridescens*	Mountain MD	SRR	100%	5	--	2
			TOTAL =	76	15	13

Skin secretions were elicited by acetylcholine injection or via massage and then analyzed via proteomics. Skin samples were collected and peptide profiled using transcriptomics. Asterisk (*) indicates skin secretions were collected via massage and then skin samples were collected for transcriptomics; all other samples were independent.

**Fig. 1:**
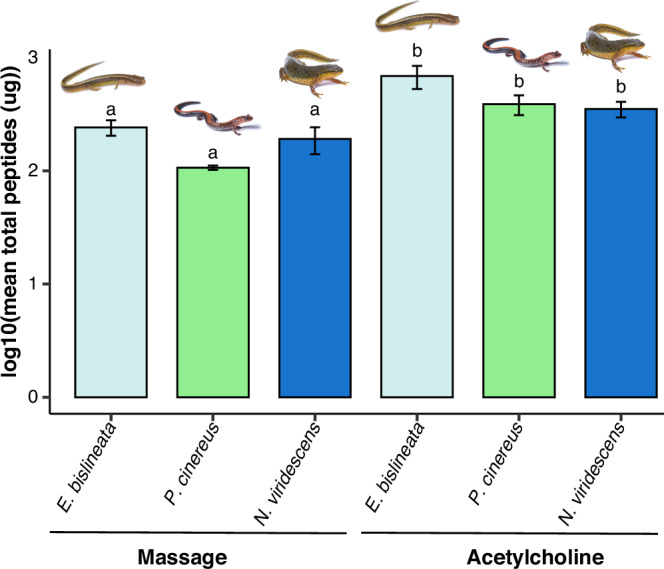
Bar plot of mean total peptide content log_10_ transformed per treatment. Letters above bars indicate significance between groups with acetylcholine injections (“b”) causing greater total peptide content recovery compared to massage treatment (“a”), regardless of species. Error bars represent SE. Photo credit: Brian Gratwicke.

Acetylcholine injection led to greater amounts of total peptides recovered (*n* = 76, mean ± SE = 458.6 ± 58.1 μg) than the massaged treatment (*n* = 15, 164.5 ± 26.9 μg), regardless of salamander species (Fig. 1, ANOVA, Treatment *F*_1, 84_ = 6.55, *p* = 0.01). Both treatments yielded greater total peptide content than negative control peptide extractions (*n* = 10, 49.5 ± 15.8 μg; ANOVA, *F*_2, 98_ = 21.54, *p* < 0.001, Tukey HSD *p* < 0.03).

### Multi-omic tools fueled AMPs discovery

We conducted skin transcriptomics on whole skin samples (*n* = 13; Table 1) and proteomics on skin secretion samples (*n* = 91; Table 1) to identify candidate AMPs and assigned AMPs to AMP families (Supplementary Data 1) based on sequence similarity to the InterPro database^38^ and five published AMP databases (APD^39^, DADP^40^, DBAASP^41^, dbAMP^42^ and DRAMP^43^). We designated the AMP families as (1) definitive AMPs with no other known function or (2) definitive AMPs with additional non-immune functions. For instance, a 39-residue long antimicrobial peptide (Buforin I) is derived from the C-terminus of Histone H2A^44^ (a well-conserved protein involved DNA packaging). Similar AMP families exist (Supplementary Data 1) and therefore we designate peptides matching to known AMPs as candidate AMPs.

**Fig. 2:**
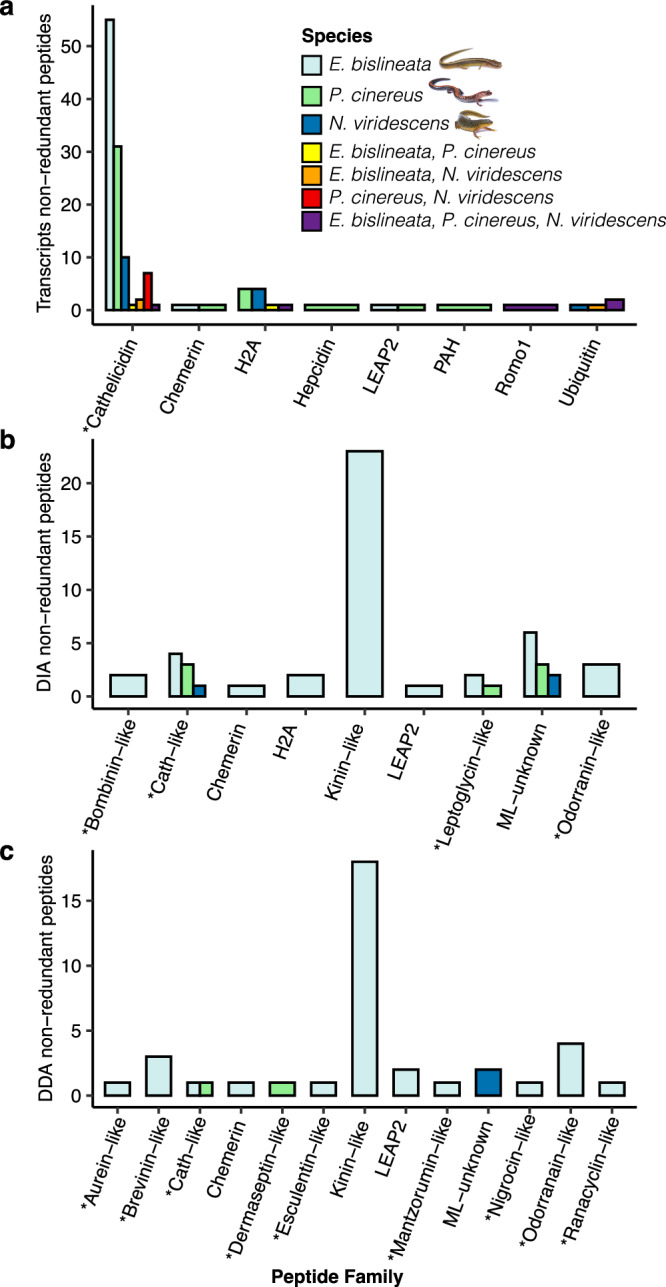
Bar plots of classification of non-redundant peptides showing homology to antimicrobial peptides. Cathelicidin peptides were commonly detected in transcriptomic data (**a**), whereas Kinin-like peptides were commonly detected in proteomic data using DIA (**b**) and DDA (**c**) mass spectrometry methods. Peptide families known to only serve antimicrobial function (and not other non-immune functions) are noted with an asterisk.

**Fig. 3:**
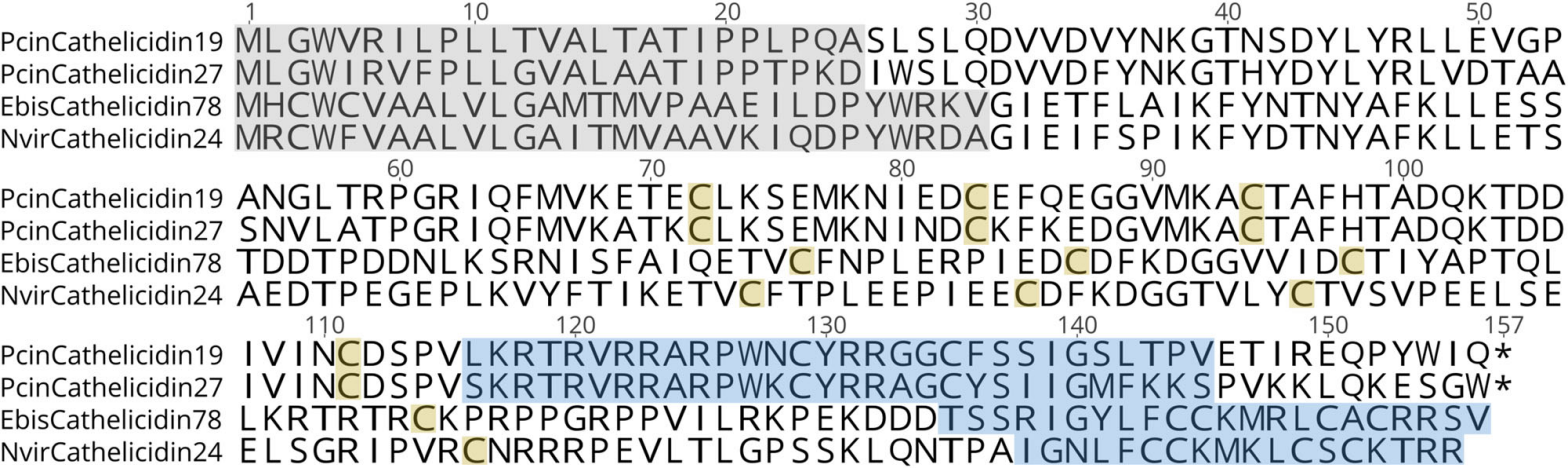
Cathelicidin pre-propeptide sequence diversity in three unique cathelicidin-like peptides. Gray boxed residues represent the signal peptide (pre) region. Highlighted are the four conserved cysteine residues present in the cathelin domain (pro) region. Blue boxed residues represent the predicted mature antimicrobial (peptide) region. Asterisk (*) notes continuation of the sequence. The two newly identified AMPs were synthesized from PcinCathelicidin19 and 27, known as Pcin-CATH3 and Pcin-CATH5 as mature AMPs.

We examined the diversity of candidate AMPs discovered using whole skin transcriptomics. A total of 279 amino acid sequences were predicted to belong to an AMP family, which clustered into 150 non-redundant AMP-like peptides (Fig. 2a, Table 2, Supplementary Data 2). Every individual salamander had transcriptionally active candidate AMP genes (Table 2, Supplementary Data 3–5). Cathelicidin peptides were the most abundant and detected in all species, ranging from 86 to 422 residues long (Figs. 2a, 3, Supplementary Data 2). Across all three salamander species, sequences from five candidate AMPs were shared, which included one Cathelicidin (Fig. S1), one Histone H2A (Fig. S2), one Reactive oxygen modulator 1 (Romo1), and two Ubiquitin peptide sequences (Fig. 2a).

**Table 2:** Counts of non-redundant candidate AMP sequences per discovery method and species

	Transcriptomics no. of AMP-like sequences	Proteomics no. of AMP-like sequences (DIA, DDA)
*E. bislineata*	67	59 (44, 34)
*P. cinereus*	53	9 (7, 2)
*N. viridescens*	30	5 (3, 2)
TOTAL:	150	73 (54, 38)

For proteomics, non-redundant within DIA and DDA methods are listed within parentheses

We examined the diversity of candidate AMPs discovered using liquid chromatography-tandem mass spectrometry (LC-MS/MS) proteomics on peptides harvested from salamander skin secretions (Fig. 2b, c). Data were collected as both data independent analysis (DIA) and data dependent analysis (DDA). We detected peptides in three individuals from the massaged treatments (*n* = 15; 20%) and in 28 individuals from the acetylcholine treatment (*n* = 76; 37%) (Supplementary Data 6), with markedly more peptides identified in *E. bislineata* compared to the other two salamander species (Table 2). A total of 420 and 174 amino acid sequences, for DIA and DDA, respectively, were predicted to belong to an AMP family, which clustered into 54 and 38 non-redundant AMP-like peptides, for DIA (Fig. 2b; Supplementary Data 7) and DDA (Fig. 2c; Supplementary Data 8), respectively. Kinin-like peptides were the most common but detected only in *E. bislineata*. Cathelicidin-like peptides were detected in all species using DIA. Eleven (DIA) and two (DDA) peptides were classified as putative AMPs from machine learning with *ampir* (ML_unknown). More peptide families (but fewer peptide sequences) were detected with DDA, with eleven peptide families detected compared to seven peptide families detected with DIA (Table 2; Fig. 2). Twelve candidate AMPs found in DIA analyses (1 Chemerin, 8 Cathelicidins, 2 H2A, and 1 liver-expressed antimicrobial peptides [LEAP2]) and two peptides found in DDA analyses (1 Chemerin and 1 Cathelicidin) were also detected in whole skin transcriptomic analyses. Only one Chemerin AMP-like sequence was detected using all methods.

### Skin AMP composition linked to skin bacterial composition

**Fig. 4:**
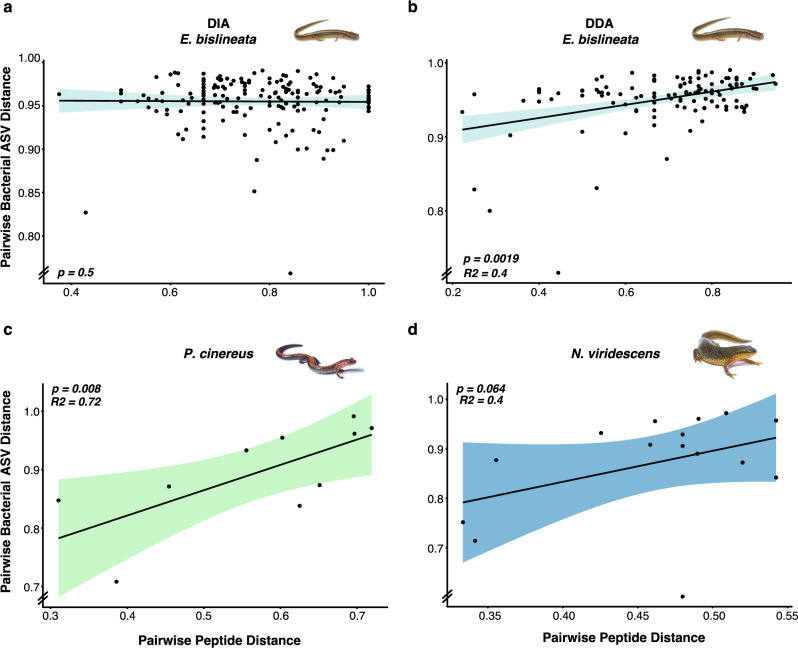
Scatterplots showing relationship of pairwise Jaccard distances of bacterial ASV composition between pairwise Jaccard distances of peptide composition. Relationships shown for E. *bislineata* using **a** DIA and **b** DDA peptide data, and for **c** *P. cinereus* and **d** *N. viridescens* using transcriptomic peptide data.

Candidate AMP composition generally predicted skin bacterial composition. For *E. bislineata*, DDA peptide composition predicted skin bacterial composition in both measures (Jaccard-Jaccard: *p* = 0.0019, *r* = 0.40; Bray-Bray: *p* = 0.0072, *r* = 0.33), but not from DIA discovered peptides (Figs. 4, S3). For *P. cinereus*, transcriptome-derived peptide composition predicted bacterial composition for Jaccard-Jaccard (*p* = 0.008, *r* = 0.72) and trended towards predicting composition for Bray-Bray (*p* = 0.075, *r* = 0.32) (Figs. 4, S3). For *N. viridescens*, transcriptome-derived peptide composition trended towards predicting bacterial composition (Jaccard-Jaccard, *p* = 0.064, *r* = 0.40, Bray-Bray *p* = 0.09, *r* = 0.49) (Figs. 4, S3). Correlations between individual bacterial amplicon sequence variants (ASVs) and peptides (Figs. S4–6) revealed multiple relationships in *E*. bislineata (DDA: 9 negative relationships; DIA: 5 positive, 17 negative), in *P. cinereus* (transcripts: 80 positive, 49 negative) and in *N. viridescens* (transcripts: 7 positive, 8 negative). To note, were bacterial-peptide relationships containing putatively Bd-inhibitory bacterial ASVs (matching a Bd-inhibitory 16S rRNA database) and that were observed in multiple salamander species, which included: (i) *Pseudomonas* ASV42 that showed positive relationships with two Kinin-like peptides (Kinin-like 31 and 32) in *E. bislineata* and one Cath-like peptide in *P. cinereus*, (ii) *Aeromonas* ASV59 that showed positive relationship with the same two Kinin-like peptides (Kinin-like 31 and 32) in *E. bislineata*, but showed negative relationships to a Cath-like peptide in *N. viridescens*, (iii) *Pseudomonas* ASV551 that showed positive relationships in *P. cinereus* with two Cath-like and two Buforin(H2A)-like peptides and negative relationship with one Cath-like in *P. cinereus* and two Kinin-like peptides in E*. bislineata*, and (4) *Stenotrophomonas* ASV113 that showed negative relationships with two Kinin-like peptides in *E. bislineata* and both positive and negative relationships in *P. cinereus* with Cath-like and Ubiquitin-like peptides (Figs. S4–6).

### Crude and synthesized peptides showed limited activity against Bd

**Fig. 5:**
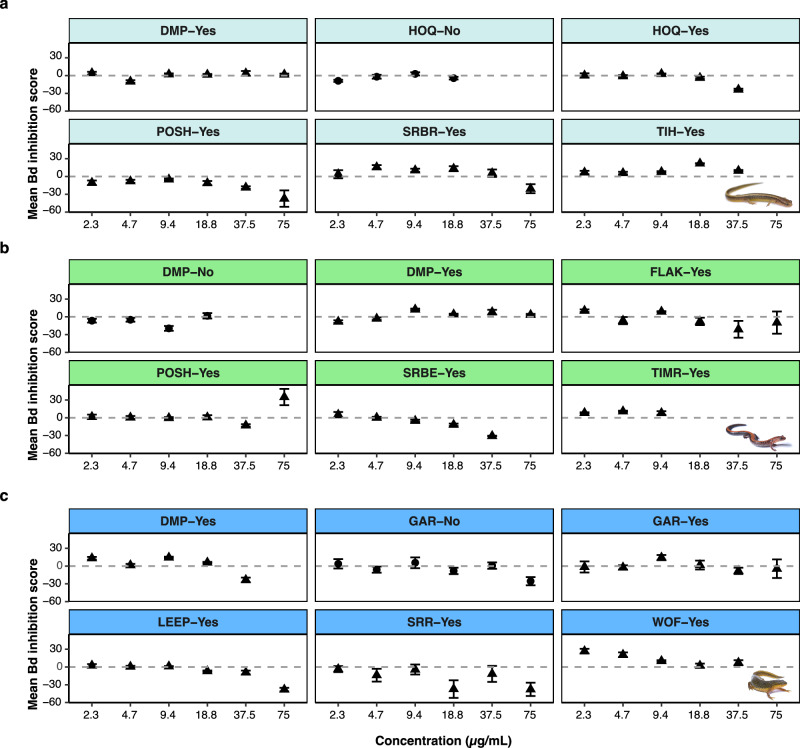
Mean inhibition scores (± SE) of crude peptides against Bd across concentrations. We observed mild to no inhibition of crude peptides against Bd for **a** *E. bislineata*, **b** *P. cinereus* and **c** *N. viridescens*. Peptides collected from the same species, site and peptide elicitation method, acetylcholine (▴) or massage (•) were combined to be challenged against Bd in 96-well plate assays.

Crude peptides from 16/18 composite samples (composite of species-site-injection method) showed mild inhibition of Bd growth at one concentration or more (Fig. 5, Supplementary Data 9). The highest Bd inhibition was 35% from *P. cinereus*-POSH-Yes at 75 μg/mL. Three samples, from different species and sites, showed mild Bd inhibition at all concentrations tested (*E. bislineata*-TIH-Yes, *P. cinereus*-TIMR-Yes, *N. viridescens*-WOF-Yes).

From the -omic data, we identified and synthesized 20 peptides to be used in challenge assays with pathogens (Supplementary Data 10). Two synthetic peptides showed mild inhibition of Bd growth (Fig. S7, Supplementary Data 11). Both Cathelicidin-like peptides were synthesized based on proteomic sequences of *E. bislineata* (PrAd-Eb12 and PrMb-Eb19). Only PrAd-Eb12 showed mild Bd inhibition at an average of 1–9.5% across concentrations down to 31.25 μg/mL. The other synthetic peptide, PrMb-Eb19, only showed mild Bd inhibition at 35% at 500 μg/mL concentration. Given the mild Bd inhibition, we did not consider these peptides to be antimicrobial.

### Synthesized peptides showed activity against human pathogens

Two synthetic peptides of twenty showed inhibition of multiple strains of MDR human pathogens (Table 3, Supplementary Data 12). Both Cathelicidin peptides (Fig. S8) were synthesized based on transcriptomic sequences of *P. cinereus* (Tr-Pc3 and Tr-Pc5). Cathelicidin Tr-Pc3, hereafter Pcin-CATH3, showed moderate inhibition of three strains of *Acinetobacter baumannii*, one strain of *P. aeruginosa* and one strain of *E. coli*, but at higher MICs than the human Cathelicidin LL-37 and antibiotic Levofloxacin. Cathelicidin Tr-Pc5, hereafter Pcin-CATH5, showed comparable MICs to LL-37 and Levofloxacin for *Acinetobacter baumannii* strains, particularly 5075, as well as showing moderate inhibition of one *P. aeruginosa* strain and two *E. coli* strains. No HepG2 cytotox was observed at concentrations up to 5.25 μg/mL for LL-37, Cath-Pc3 and Cath-Pc5. Given the moderate to strong inhibition of multiple MDR bacteria, we consider these peptides to be new AMPs. Peptide names were given according to binomial nomenclature acronym of the species followed by “CATH” indicating cathelicidin. The peptide sequences were Pcin-CATH3 (N-LKRTRVRRARPWNCYRRGGCFSSIGSLTPV-C) and Pcin-CATH5 (N-SKRTRVRRARPWKCYRRAGCYSIIGMFKKS-C).

**Table 3:** MIC values (μg/mL) for the two new AMPs discovered (Pcin-CATH3 and Pcin-CATH5) from *P. cinereus* transcriptomic data

	*E. faecium*		*S. aureus*		*K. pneumoniae*			*A. baumannii*			*P. aeruginosa*			*E. cloacae*		*E. coli*	
Compound ID	19434	49224	25923	43300	700603	13883	4640	17978	19606	5075	PA01	27853	5519	13047	35549	35218	43888
Pcin-CATH3 (Tr-Pc3)	>128	>128	>128	>128	>128	>128	>128	64	64	16	32	>128	>128	>128	>128	>128	64
Pcin-CATH5 (Tr-Pc5)	64	64	>128	64	>128	>128	>128	8	8	4	16	>128	>128	>128	>128	32	16
LL-37	32	32	>128	>128	32	4	64	4	2	4	4	8	16	64	4	8	8
Levofloxacin	4	2	0.25	0.25	0.5	0.25	32	0.25	0.25	4	8	0.5	32	0.25	0.25	0.25	0.25

LL-37 and Levofloxacin were used as positive controls. Strain IDs for the ESKAPEE panel are indicated below each bacterial species.

## Discussion

Our study is the first to identify diverse AMPs and their relationship with diverse skin microbiomes across multiple vertebrate species. We identified a high diversity of candidate AMPs in three Appalachian salamander species with some overlap in peptide families, but little overlap in candidate AMPs between salamander species. Transcriptomics of whole skin performed better in discovery of candidate AMPs than proteomics of minimally invasive skin secretions for *P. cinereus* (red-backed salamanders) and *N. viridescens* (eastern newts), whereas for *E. bislineata* (two-lined salamander) a highest diversity of candidate AMPs was detected using both -omic methods. In future work, toe clips and tail clips could also be explored as non-lethal sampling methods for AMP discovery. Across salamander species and AMP discovery methods, skin peptide composition was generally correlated with skin bacterial composition suggesting bi-directional interactions between skin peptides and the skin microbiome. Such peptide-microbial crosstalk may explain patterns of phylosymbiosis in Appalachian salamanders^45^. In these salamander populations, crude and synthesized skin peptides showed minimal antimicrobial activity against Bd, suggesting that other immune properties (e.g., skin microbiome, immune cells) may be more influential in directly impacting salamander-Bd interactions, whereas AMPs may indirectly shape these responses through direct effects on the skin microbiome. We identified two synthesized peptides that killed MDR bacteria, which we named as two newly discovered AMPs—Pcin-CATH3 and Pcin-CATH5—increasing the number of described salamander AMPs to eleven^17^. Our findings contribute to understanding the microbial-immune interface in amphibians with broader applicability to both basic and applied scientific inquiry.

The three Appalachian salamander species produced a diverse repertoire of candidate AMPs in their skin. However, the discovery of AMPs in salamanders was challenging. We initially turned to standard methods used in discovery of AMPs in frogs, using a neurotransmitter injection to stimulate peptide release^6,23,46^. In frogs, the neurotransmitter used to elicit peptide release is norepinephrine. However, in salamanders, the neurotransmitter acetylcholine was suggested to elicit peptide release^47^, albeit with the caveat that exposure method matters, in which soaking is just as effective as massage^48^. Here, we show that acetylcholine injections led to higher peptide yields from harvest skin secretions and greater peptide diversity discovered using proteomics compared to massaged salamanders in *E. bislineata*. However, even with acetylcholine injections, few peptides were detected from skin secretions with proteomic methods in *P. cinereus* or *N. viridescens*. In the field, the dose we used for *E. bislineata* and *P. cinereus* generally elicited an observable physiological response, but this was rarely observed in *N. viridescens* suggesting species-dependent responses and potential for more AMPs being in eastern newts (*N. viridescens*) if a higher dose were used. For large-bodied salamanders such as hellbenders, no injections can recover high quantities of peptides from single individuals due to the high surface area^49,50^. For small-bodied salamanders, such as the species studied here, AMP discovery may be most effective by combining skin secretions from >15 individuals for proteomics and combining with skin transcriptomics.

Most candidate AMPs and peptide families were uniquely discovered between the -omic methods. Perhaps this is not surprising as we used different sampling techniques (whole skin versus skin secretion collection) between the two -omic methods. However, even if we used the same sample type transcriptomics and proteomics methods are commonly reported to differ in protein discovery^51,52^ due to factors such as: (i) post-transcriptional modification (e.g., splicing) affecting mRNA that affects protein synthesis, (ii) post-translation modifications (e.g., phosphorylation) that affect protein activity and stability, and (iii) protein half-life that vary from minutes to years. In our dataset, notably, a variety of Cathelicidins genes were commonly detected in whole skin transcriptomic data in all species. Cathelicidins were detected less frequently in the skin secretion proteomics suggesting that (i) those genes were actively making larger precursor Cath-like proteins, but post-translational modification to an active peptide form were occurring less frequently (perhaps due to their potential detrimental effect on vertebrate cells^53^, (ii) Cathelicidins were primary constrained to circulation within skin blood vessels^13,54^ and/or proteomic analysis had technical limitations given low peptide yields from skin secretions. However, all Cathelicidins detected with proteomics were also found in transcriptomics (it was just far fewer were detected using proteomics) showcasing that these candidate AMPs are being produced by the salamanders and stored in high amounts for detection in skin secretions. On the other hand, we found several peptide families in skin secretion proteomics, not detected in whole skin transcriptomics. These candidate AMPs may (i) be made, then build up in the glands where continual expression is not needed or (ii) not have been detected given our transcriptomic sequence coverage at 40 M reads per sample. Notably, Bombinin-like, Odorranain-like, and Leptoglycin-like peptides were present in *E. bislineata* and are each known families of amphibian AMPs^55–57^. One Chemerin-like candidate AMP was detected in *E. bislineata* across proteomic (DIA, DDA) and transcriptomic analyses and showed a negative relationship with a putatively Bd-inhibitory *Enterobacter* sp. (ASV 1280) highlighting it may be abundant and influential in microbial interactions. In humans, a chemerin AMP showed relationships to microbial-immune outcomes^58,59^ and warrants future study in amphibians, such as the African-clawed frog *Xenopus laevis*^60^. To our knowledge, our study is the first to integrate transcriptomic and proteomic data in amphibian species for AMP discovery.

We found evidence that the diverse repertoire of AMPs in Appalachian salamander skin was interconnected with skin bacterial composition. Studies in insects, cnidarians and humans provide compelling evidence that AMPs can influence microbiome composition^2–5^ and that each animal species can produce unique AMP profiles that can maintain species-specific microbiomes^2^. To our knowledge, a few studies have examined AMP-microbial interactions in amphibians, but only in frogs^16,32,37,61^. In assays using frog-derived AMPs and culturable skin bacteria, AMPs generally enhanced symbiotic bacterial growth showcasing that AMPs can also serve a pro-microbial role^16,32^. Using molecular approaches, frog-derived AMPs correlated both positively and negatively with frog skin bacterial taxa^37^, as we also found in our current study in salamanders, suggesting that skin peptides may act as a filter for environmentally-derived bacteria in amphibians. Studies of AMP-microbiome interactions are still rare in the literature particularly in vertebrates, and more comparative work such as presented here will be key to making advancements in understanding broad patterns of microbiome community assembly^45,62^ and informing models on the evolution of host AMPs and symbiont resistance^63^.

Novelly, we found AMP composition being linked to skin microbiome composition across multiple salamander species. Appalachian salamanders show a pattern of phylosymbiosis in their skin microbiomes, in which the skin bacterial communities on the salamanders change in parallel with the evolutionary history of the salamanders^45^. We hypothesize that both the host environmental microbiome is influencing the AMPs being produced and the AMPs being produced are influencing the assembling of the skin microbiome from environmental sources (e.g., as in *Drosophilia*^5^) which are acting as selective pressures in parallel with host evolution over both ecological and evolutionary time frames. As in other systems, experimental manipulations and large scale comparative phylogenetics are essential to identify key players and drivers of these interactions^2,5^.

We identified a few target peptides and bacteria that would be useful for future study. One Cathelicidin peptide (Fig. S1) was found in all three salamander species and may be a useful target for comparative studies, such as targeted knock-outs to determine the effects on the microbiome (e.g., as in beetles^3^). Additionally, some of the newly discovered peptides displayed both positive and negative interactions with individual bacterial taxa showcasing a dynamic anti-microbial and pro-microbial nature of these candidate AMPs. For instance, four bacterial ASVs (*Pseudomonas* spp. ASV42 and ASV551, *Aeromonas* ASV59, and *Stenotrophomonas* ASV113) showed multiple positive and negative relationships with peptides and were detected in at least two of the salamander species. *Stenotrophomonas* ASV113 matched at 100% sequence similarity to a Bd-inhibitory isolate *Stenotrophomonas rhizophilia* THA2.2 we previously isolated from *P. cinereus*^64^, was identified as a hub bacteria in skin microbiome networks for *P. cinereus* and *N. viridescens* in different populations^65^ and was the focus of our recent study examining its effects on the gene expression response of African-clawed frogs *X. laevis*^66^. In Madison et al.^66^, *S. rhizophilia* was also a common symbiont of *X. laevis* and its inoculation onto *X. laevis* skin impacted other components of the innate immune system including FCN2 (binds PAMPs), CFB (part of complement activation), and IL12B (an interleukin). Our findings pave the way to further examine these interactions and their influence on host ecology and evolution.

Two pathogens, Bd and Bsal, are of particular concern in Appalachia, USA, the global hotspot of salamander biodiversity. It is likely Appalachian salamanders have been exposed to Bd in their environment for 50+ years^67^, with little evidence of host mortality driven by Bd infection in recent decades^18,68^. Whereas, Bsal has yet to reach Appalachia or the Americas^19^ and declines, extirpations and extinctions are a major concern for salamanders in Appalachia^69,70^. If Bsal or a hypervirulent Bd strain were introduced into the eastern US understanding of the relationships between AMPs and microbiomes can inform conservation strategies^29^. Based on Bd prevalence in the field and experimental Bsal infections, *E. bislineata* and *P. cinereus* are relatively resistant to Bd and Bsal infection^65,69,70^, while *N. viridescens* is commonly infected with Bd and likely tolerates infection^65^, whereas they are highly susceptible to Bsal^19,69^, with limited innate defenses^71^. Here and in our previous study using non-target collection of crude secretions (mucosomes^65^) we found mild killing ability of Bd and Bsal, which was similar to another study^71^. In this current study, we did not recover peptide profiles in adequate sample sizes to test for relationships between Bd infection and candidate AMP profiles. Most Bd-positive individuals we sampled were from the species *N. viridescens*, a species commonly known to be infected with Bd, but our recovery of candidate AMP profiles was limited in this species (proteomic *n* = 3 Bd+; transcriptomic *n* = 4 Bd+, *n* = 2 Bd−). There appears to be some evidence for mild selection for defensive AMPs in our previous study on *N. viridescens*^65^ and in the frog species, *Lithobates sphenocephala*^61^ in North America, but not at the magnitude observed in regions (i.e., Panama) where Bd-induced selection in AMP profiles has been observed due to a Bd epizootic event^23^. In our current study, it is possible that we found limited Bd inhibition because the AMPs from these three salamander species (i) are not optimized for Bd inhibition compared to other species^23^, (ii) were not in high enough concentrations^49^, and/or (iii) reflect sampling during a season when AMP Bd inhibition effectiveness was low^71^. We hypothesize that in Appalachian salamanders (where Bd is in an enzootic state) a defensive microbiome, derived by selectively filtering environmental microbes through AMP composition, plays a stronger role in Bd resistance than direct effects of AMPs on Bd.

Not only are AMPs of interest in wildlife microbial ecology and conservation, but they also show promise for development into agents with therapeutic potential in human medicine^27,36,72^. We discovered two new Cathelicidin AMPs identified in the skin transcriptome of *P. cinereus* with killing activity against *A. baumanni*, *P. aeruginosa* and *E. coli*. Cathelicidins are widely known AMPs and found across the vertebrate tree of life^54^. Two other Cathelicidins have previously been identified in Asian salamanders; one Cathelicidin (TK-CATH) showing hemolytic, antioxidant, anti-inflammatory and wound-healing properties from Kweichow newt, *Tylototriton kweichowensis*^73^ and another (AdCath) showing antimicrobial activity from Chinese giant salamander, *Andrias davidianus*^12^. We found no cell-killing activity by these two new peptides and look forward to further pursuing their application in human therapeutics.

As AMPs in diverse multicellular organisms were discovered, Zasloff predicted in 2002^74^ that every species harbors a unique and specific collection of AMPs tuned to defend the organism against microorganisms. This prediction has been supported with decades of research since^75^. With emergence of new methods in AMP discovery, great strides can be made in better understanding their diversity, function and application across all domains of life^76,77^. We now suggest that our attention should turn to how these unique and specific collection of AMPs interact with the microbiome of the host and their role in establishing and maintaining these critical host-microbial symbioses. Both AMPs^23,26,27^ and host microbiomes can be modified rapidly^78^, which may allow hosts to rapidly adapt to changing environments^25^. There is great potential for engineering the microbiome for a myriad of applications, from reducing wildlife disease^79^, to improving crop yields with biofertilizers^80^, to novel immunization strategies for humans^81^. Insight into the AMP-microbial interface will improve effectiveness of microbiome engineering and our understanding of how balanced and sometimes imbalanced interactions impact host resilience to environmental change and eco-evolutionary processes more broadly. Our study paves the way to use comparative vertebrate species systems to understand AMP-microbiome interactions.

## Methods

### Field sampling in Appalachian Mountains

We sampled 100 adult salamanders from three species, northern two-lined salamander (*E. bislineata*: *n* = 25), red-backed salamander (*P. cinereus*: *n* = 36) and eastern newt (*N. viridescens*: *n* = 39), at 12 sites within three localities across a 150 km range in the Allegheny Plateau and Ridge & Valley physiographic provinces of the Appalachian Mountains, USA^45^ (Table 1). We had IACUC approval from NZCBI (#18–19) and scientific collection permits (MD DNR 57633, VA DWR 067393, USFWS MA93679B). All equipment and footwear were sterilized with 10% bleach between localities.

Upon capture, each salamander was rinsed with sterile water and swabbed 25 times with a rayon swab (MW-113)^65^. After swabbing, the salamander was randomly assigned to either an acetylcholine injection treatment (*n* = 76), a “massaged” treatment (*n* = 15) or a transcriptomic treatment (*n* = 13) to examine effects of sample collection on peptide discovery (Table 1). We chose to use acetylcholine injections because neurotransmitter injections have been shown to stimulate peptide release in frogs^6,23,46^. However, in salamanders, the neurotransmitter acetylcholine has been shown to be more effective in eliciting peptide release than norepinephrine, which is used in frogs^47^. We chose to use a massage treatment also, as a study be Pereira et al.^48^ found that a massage treatment was just as effective in peptide recovery as soaking salamanders in acetylcholine, and therefore we wished to verify that acetylcholine injections would lead to greater peptide recovery than massage alone.

For the salamanders treated with acetylcholine, we used a sterile insulin needle to inject acetylcholine (2.5 μmoles/gram body weight) based on body mass, added MilliQ (Ultrapure Type 1) water to the bag, and then allowed them to rest for 10 min. For the massaged treatment, we added MilliQ water to a WhirlPak bag containing a salamander, tapped the salamander’s lower dorsum for 5 min and then allowed them to rest for 5 min. For small-bodied salamanders (<2 grams: all red-backs, most two-lined salamanders), they were soaked in 5 mL of water and if assigned acetylcholine treatment, were injected with 50 μL/gbw of 0.05 M acetylcholine. For medium-bodied salamanders (<2 grams: some two-lined salamanders, all newts), they were soaked in 10 mL of water and if assigned the acetylcholine treatment, were injected with 25 μL/gbw of 0.1 M acetylcholine. Different volumes of water were used so that the salamander was just fully submerged in water. Acetylcholine (Sigma Chemical Co., Cat No. A-2661) was diluted with 1X amphibian phosphate buffered saline and filter-sterilized prior to use and different concentrations were used to keep the injection volume between 25 and 150 μL of acetylcholine solution. After the water bath, salamanders were removed from the WhirlPak bag and the remaining aqueous solution was poured into a 15 mL Falcon Tube and put directly onto dry ice, and then stored at -80C until processing. In the field, we randomly collected four WhirlPak bag and MilliQ water negative controls for peptide extraction. We noticed short term sedation from acetylcholine injection in a small percentage of red-backed salamanders and two-lined salamanders, but never in any eastern newts. For future studies lower concentrations of acetylcholine should be considered for use in Plethodontid salamanders. After sample collection, most salamanders were released at the site of capture (*n* = 87). For transcriptomics, 13 salamanders were humanely euthanized by applying 20% benzocaine to upper ventrum and lower ventrum skin samples (5 mm × 5 mm) were preserved in RNAlater (Table 1).

### Transcriptome assembly

RNA was extracted from skin samples (*n* = 13, Table 1) and then sequenced to ~40 M reads per sample with PE150 on an Illumina HiSeqX10 by Omega Bioservices. Total RNA was isolated with E.Z.N.A.® Total RNA Kit (Omega Bio-tek). Libraries were prepared from polyA mRNA with input of 500 ng high quality total RNA (RIN > 8) using Illumina TruSeq Stranded mRNA Library Prep Kit. We conducted transcriptome assembly by broadly following recommendations by MacManes^82^. Pre-assembly reads were corrected using RCorrector using default settings, and adapters were trimmed using Trimmomatic. rRNA reads were filtered out by mapping reads to the SILVA database using bowtie2. Reads were assembled using Trinity (k-mer = 25), rnaSPAdes (k-mer = 55 and 75), and BinPacker (k-mer = 25). These assemblies were combined into a non-redundant assembly per salamander species and ORFs were translated using EvidentialGene^83^. Post-assembly QC was done with BUSCO and TransRate.

### Skin peptide extraction and analyses

To extract peptides from skin secretions, we followed the C-18 Sep-Pak cartridge (Waters Corp, Cat No. WAT 020515) and buffer extraction methods by Pereira & Woodley^49^ using a vacuum manifold. We extracted peptides from 91 salamander water samples, four field negative controls, and six lab negative controls. A total of 20 mL of peptides were eluted and concentrated in individual 2 mL microcentrifuge tubes with a Speed-Vac concentrator. Peptide content of one aliquot was resuspended with MilliQ (Ultrapure Type 1) water and total peptide content was estimated using a Micro BCA Protein Assay Kit Assay Kit (Thermo Scientific, Rockford, IL, USA) using 589 nm absorbance readings. Bradykinin was used as the standard (Sigma Chemical Co) and all samples were run at two dilutions in duplicate. The BCA curve is 2nd order polynomial, and we used the standards’ absorbances and the Wolfram Alpha widget (https://www.wolframalpha.com/widgets/) “rearrange it” solver (to rearrange for x to determine the concentration) and then back calculated to determine total peptide concentration in the total 20 ml of eluted peptides. If the estimates between two dilutions were greater than 10X (*n* = 2) or only one dilution was quantifiable (*n* = 15), the sample was re-run and for 10X differences the outlier was removed after re-quantification.

To determine if peptide content recovery was affected by acetylcholine vs massaged treatments, we used an ANOVA with log-transformed peptide amount as the response and treatment, species and their interaction as the explanatory variables. Tukey HSD test was used in post hoc analyses. We also verified that biological treatments were recovering more peptides than negative controls and used an ANOVA with log-transformed peptide amount as the response and treatment (acetycholine, massage, control) as the explanatory variable.

### Proteomic methods and analyses

Dried peptides were resuspended and analyzed using LC-MS/MS. Dried peptides were resuspended in 30 µL of 0.1% formic acid at ~2 µg/µL except for samples with less than 30 µg of total peptide using an Opentrons OT-2 liquid handler and covered for subsequent liquid chromatography (UltiMate 3000 UPLC). From individual samples of peptides, 10 µg were injected onto ThermoScientific Acclaim PepMap 100 trap columns (100 µm i.d. x 2 cm, 5 µm particle size) and separated on a ThermoScientific Acclaim PepMap RSLC analytical column (75 µm i.d. x 25 cm, 2 µm particle size) with the following gradient: 0–5 min 2%B, 5–6 min 2–13%B, 6-28 min 13–24%B, 28–39 min 24–36%B, 39–41 min 36–80%B, 41–46 min 80%B, 46–47 min 80–2%B, 47–60 min 2%B. Solvent A was 0.1% formic acid in water and solvent B was 0.1% formic acid in acetonitrile. Separated peptides were nanosprayed at 1.85 kV into an Orbitrap Elite mass spectrometer.

Data were collected as both DIA and DDA. For DIA, a precursor mass scan (MS1) was collected at 60,000 resolving power from 375 to 2000 m/z. From m/z 350 to 785.5 a 30 m/z isolation window with 1 m/z overlap was used to collect fragmentation spectra (MS2). From m/z 769.5 to 1082.5, a 105 m/z isolation window was used with 1 m/z overlap. HCD charge state was set to 2. All fragmentation used 30% normalized collision energy (NCE) higher energy collision dissociation (HCD). Isolation widths follow Rüther et al.^84^. For DDA, MS1 was collected from 375 to 2000 m/z at 60,000 resolving power. A top 10 method was used with the following parameters: 15,000 resolving power, 30% NCE HCD, default charge state 2, 0.1 ms activation time, fixed first mass at 100 m/z. Resultant RAW files were searched with MetaMorpheus 0.0.320 using AMP databases generated from transcriptomes derived from these species or a single database of unique AMPs from five AMP databases (see next subsection). Summed intensities for peptides and peptide spectral matches (PSMsum) were calculated in R. We also analyzed samples pooled together by species and site and those results re-capitulated what we present here.

### AMP identification

Candidate AMPs were identified and extracted from transcriptomes and proteomes via two methods. In method 1, we searched pFAM (now InterPro) for the search terms “Antimicrobial peptide”, “Antifungal peptide”, “Antibacterial peptide”, “defense” and “defence”, and manually checked protein sequence annotation to verify AMP-like sequences. Then, we used eggNOG-mapper to search the custom pFAM database for matches and restricted the taxonomic scope to animals only. In method 2, we used USEARCH to identify hits to a composite database of the five published AMP databases (APD^39^, DADP^40^, DBAASP^41^, dbAMP^42^ and DRAMP^43^) and extracted only AMPs that were labeled as being derived from amphibians. Match criteria were set at identity > 95%, target coverage ≥ 90% and *e* ≤ 0.0001. We also looked for potential novel AMPs with machine learning using ampir^85^; more than 950 AMPs per species were identified in transcriptomic data and we chose to only include those in future analyses if they also matched to proteomic data as expressed peptides. Given our databases used to identify AMPs were restricted to animals/amphibians, we made the assumption that the candidate AMPs we identified were host derived.

To identify AMP overlap between individuals and species, candidate AMPs were merged and clustered using CD-HIT (clustering threshold 95% identity, 90% shorter sequence length threshold). Peptide clusters were formed from transcriptomes (Supplementary Data 2*)*, proteomics DIA (Supplementary Data 7) and proteomics DDA (Supplementary Data 8) separately and the representative sequence, the longest sequence in each cluster, was used. For transcriptomic data, we had the pre-propeptide sequence (i.e., precursor protein) for candidate AMPs and therefore were able to define AMPs as belonging to specific families based on structural features (e.g., cathelin domain for Cathelicidin) and/or sequence homology. For proteomic data, we only had the mature peptide sequence and lack the sequence information to place them into definitive AMP families and therefore refer to them as family-like (e.g., Aurein-like) to the specific family of AMP that the sequence matched at identity >95%, target coverage ≥ 90% and *e* ≤ 0.0001.

### Pathogen and microbiome molecular methods and analyses

Genomic DNA was extracted from skin swabs using the DNeasy PowerSoil HTP 96 kit (Qiagen 12955) including positive (Zymo, Catalog No. D6300) and negative extraction controls. From the extracted DNA, we used qPCR for the detection of Bd, Bsal and ranavirus infection and sequenced the 16S rRNA V3-V5 gene region on an Illumina MiSeq along with positive (Zymo Catalog No. D6305) and negative controls as fully detailed in Osborne et al.^45^.

We examined correlations between bacterial composition and peptide composition and bacterial ASV abundance (measured as log10 sequence counts) and peptide abundance (proteomics = measured as log10 PSMsum; transcriptomics = measured as arcsin-transformed TPM (transcripts per million)). For proteomic-microbiome paired data, we recovered little proteomic data from *P. cinereus* and *N. viridescens*, and therefore focused analyses on *E. bislineata* using DIA (Supplemental Data 7) and DDA (Supplemental Data 8) data. *E. bislineata* microbiome data was subset to only contain the salamanders for which DIA data was obtained (*n* = 19 salamanders) and DDA (*n* = 16 salamanders). In the DIA dataset, there were 2367 bacterial ASVs and 44 putative AMPs detected. In the DDA dataset, there were 1821 bacterial ASVs and 34 peptides detected. We conducted mantel tests with Jaccard-Jaccard and Bray-Bray comparisons with 10,000 permutations. Then, we subset to do pairwise correlations to identify relationships between individual bacterial ASVs (DIA *n* = 55; DDA *n* = 44) and putative AMPs (DIA *n* = 8; DDA *n* = 7) that were present in at least 50% of individuals. We conducted both Spearman and Kendall correlations and only considered significant relationships, when both test correlations were greater than 0.4. For transcriptomic-microbiome paired data, we recovered little transcriptomic data from *E. bislineata* (given *n* = 2), and therefore focused analyses on *P. cinereus* and *N. viridescens* (Supplemental Data 4, 5). We used corset^86^ to cluster the assembled transcripts into putative genes and used those gene-level estimates. *P. cinereus* and *N. viridescens* microbiome data was subset to only contain the salamanders for which transcriptomic data was obtained (*n* = 5 *P. cinereus*; *n* = 6 *N. viridescens*). In the *P. cinereus* dataset, there were 259 taxa and 64 peptides. In the *N. viridescens* dataset, there were 128 bacterial ASVs and 32 putative AMPs detected. Mantel tests were conducted on Jaccard-Jaccard and Bray-Bray comparison with 119 (*P. cinereus*) and 719 permutations (*N. viridescens*) (max permutations possible). Then, we subset to do pairwise correlations to identify relationships between individual bacterial ASVs (*n* = 46 for *P. cinereus*; *n* = 30 for *N. viridescens*) and putative AMPs (*n* = 50 for *P. cinereus*; *n* = 32 for *N. viridescens*) that were present in at least 50% of individuals. We conducted both Spearman and Kendall correlations and only considered significant relationships when both test correlations were greater than 0.8 given the lower sample size for these two species.

### Peptide identification for synthesis

We identified 20 peptides to be synthesized for assays. These 20 peptides (Supplementary Data 10) were derived from a list of 37 candidate peptides from transcriptomic and proteomic data. We took our list of candidate AMPs to synthesize and used a variety of techniques and programs to predict antimicrobial activity and AMP-like properties. For transcriptomic data, we focused on the 119 unique cathelicidin sequences to identify peptides to synthesize using AMPA^87^ and AmpGram^88^ to identify the mature AMP region and ExPASY to predict cleavage site^89^. We examined amino acid structure in Geneious for percent hydrophobicity and charge at pH 7 to rank the likelihood of the sequences being AMPs. We also used the DBAASP antimicrobial activity prediction tool and predicting antimicrobial properties of normalized hydrophobic moment and amphiphilicity for activity against the bacterial strains that are militarily relevant (*E. coli, P. aeruginosa, K pneumoniae, S. aureus, A. baumannii*). Along with our candidate AMPs, Magainins preproprotein (NP_001081306.1) and Human CAMP (NP_004336.4) were used as positive controls. The top 20 peptides that were ranked with the best predicted activity were synthesized at 5 mg at >95% purity from Biomatik.

### Peptide challenge assays with Bd and multi-drug resistant bacteria

Bd-peptide challenge assays were conducted with the crude peptides extracted from the salamander skin secretions and with the 20 synthesized peptides. We followed methods by Pereria et al.^49^ to conduct assays with the crude peptides using Bd-GPL strain JEL404 at 1 × 10^6^ zoospores/mL. Briefly, skin peptide samples were pooled together based on species-site-injection (Table 1). Skin peptides samples were serially diluted 2-fold in MilliQ (Ultrapure Type 1) water and assayed at a concentration of 2.35–75 μg/mL based on starting peptide concentration available. For synthesized peptides, we conducted assays from 15.6 to 500 μg/ml. In both assays, we quantified percent inhibition of each well using slope of growth^90^ in which positive values indicate inhibition and negative values indicate no inhibition.

Antimicrobial susceptibility testing of the 20 synthesized peptides against 17 bacterial strains of clinical relevance in the ESKAPEE panel was conducted using minimum inhibitory concentration (MIC) assays following the broth microdilution method outlined by Parker et al.^91^. The ESKAPEE panel is comprised of fourteen American Type Culture Collection (ATCC) strains and three Multidrug Resistant Organism Repository and Surveillance Network (MRSN) bacterial strains. These strains represent at least one drug-susceptible strain and one drug-resistant strain for each bacterium to assess drug efficacy across various resistance mechanisms. This panel is comprised of the following bacterial species and strains: *E. faecium* (ATCC 19434, ATCC 49224), *S. aureus* (ATCC 25923, ATCC 43300), *K. pneumoniae* (ATCC 700603, ATCC 13883, MRSN 1320), *A. baumannii* (ATCC 17978, ATCC 19606, MRSN 959), *P. aeruginosa* (ATCC 15692, ATCC 27853, MRSN 5519), *E. cloacae* (ATCC 13047, ATCC 35549), and *E. coli* (ATCC 35218, ATCC 43888). Bacteria were grown in cation-adjusted Mueller-Hinton broth (CAMHB) to achieve a final inoculum of 1 × 10⁵ CFU/mL. CAMHB is selected for its batch-to-batch reproducibility, low levels of antimicrobial inhibitors, and ability to support the growth of most bacterial pathogens.

Briefly, lyophilized peptides were dissolved in 0.2% TFA + 0.4% BSA in water to 2.56 mg/mL, ultrasonicated for 20 min, diluted 10-fold into CAMHB, and then serially diluted 2-fold in 0.02% TFA + 0.04% BSA in CAMHB and assayed at a concentration of 0.25 to 128 μg/mL. Assays were set up using the Tecan Freedom EVO liquid handling robot and software (Tecan, Switzerland) with 50 μL of bacterial inoculum and 50 μL of peptide transferred into round-bottom 96-well microtiter plates. Each plate assay was performed in duplicate and incubated at 37 °C for 20 h without agitation. Bacterial growth was assessed using the BIOMIC V3 Image Analysis system (Giles Scientific, USA) and the MIC values were determined visually according to the Clinical and Laboratory Standards Institute (CLSI) guidelines.

For any inhibitory peptides, HepG2 (ATCC HB-8065) cytotoxicity was tested to determine half-maximal inhibitory concentration (IC_50_) of human cells. The procedure involved seeding 96-well plates with 2.5 × 10^4^ cells per well in 170 µL of culture medium, followed by an overnight incubation at 37 °C in a 5% CO₂ atmosphere. Subsequently, cells were exposed to varying concentrations of the test peptides, ranging from 5.25 µg/mL to 0.15 µg/mL, and incubated for an additional 48 h. Cell viability was assessed using the MTT assay, as previously described^92^. Absorbance measurements were obtained using a PerkinElmer EnSight plate reader. The IC_50_ values for each compound were calculated by fitting the data to a sigmoidal dose-response curve with variable slope using GraphPad Prism software. Each assay plate included three controls: (a) background control—wells without cells and without drug; (b) positive control—wells with cells and 10 µg/mL of a known toxic compound; and (c) DMSO control—wells with cells and 0.2% DMSO, the highest concentration of DMSO used in the assay, which is non-toxic to the cells.

## Supplementary information

Supplementary Information

SI-Methods&Figures-SalPeptidesRevise

TableS1-AMPfamilies

TableS2-AnnotatedTranscripts

TableS3-EbisTranscripts

TableS4-PcinTranscripts

TableS5-NvirTranscripts

TableS7-DIA-Peptides

TableS8-DDA-Peptides

TableS9-BdInhibit-Crude

TableS10-SynthesizePeptides

TableS11-BdInhibitSynthetic

TableS12-ESKAPEE-MIC

## Data Availability

We deposited demultiplexed sequence data in the NCBI SRA under BioProject ID PRJNA1039858 for the microbiome data and under BioProject ID PRJNA1157804 for transcriptomic data. Proteomic data and database search results are available at MassIVE MSV000097047.

## Code availability

Data analysis scripts are available on Github at https://github.com/CarlyMuletzWolz/SalPeptideMicro.
