# Supplementary material for: Novel antimicrobial peptides and peptide-microbiome crosstalk in Appalachian salamander skin
Source: NPJ Biofilms Microbiomes. 2025 Nov 20;11:213. doi: 10.1038/s41522-025-00837-0 (PMC12635268; doi:10.1038/s41522-025-00837-0)
Supplement: Supplementary file 1 — Supplementary Information [file 41522_2025_837_MOESM1_ESM.pdf]

Supporting information for:

'Novel antimicrobial peptides and peptide-microbiome crosstalk in Appalachian salamander skin'

Carly R. Muletz-Wolz, Julian Urrutia-Carter, Owen Osborne, Steve Kutos, Jose Meneses Montano, Joseph Madison, Brian Gratwicke, Ratanachat Racharaks, Norma E. Roncal, Randall R. Jimenez, Amy Ellison, Timothy P. Cleland

*To whom correspondence should be addressed. E-mail: craemuletz@gmail.com

This file includes supplemental data legends and figures. Supplemental data tables are provided in separate excel files.

Supplemental Data 1. **AMP families**
Table of peptide families detected, their function and associated database information.

Supplemental Data 2. **Annotated transcripts all species**
Transcriptomic-derived candidate AMPs based on non-redundant assemblies per salamander species that matched to AMPs database.

Supplemental Data 3. **Annotated transcripts *E. bislineata***
Transcriptomic-derived candidate AMPs for *E. bislineata* based on using corset to cluster the assembled transcripts into putative genes that matched to AMPs database.

Supplemental Data 4. **Annotated transcripts *P. cinereus***
Transcriptomic-derived candidate AMPs for *P. cinereus* based on using corset to cluster the assembled transcripts into putative genes that matched to AMPs database.

Supplemental Data 5. **Annotated transcripts *N. viridescens***
Transcriptomic-derived candidate AMPs for *N. viridescens* based on using corset to cluster the assembled transcripts into putative genes that matched to AMPs database.

Supplemental Data 6. **Compare DDA, DIA and injection method on AMPs discovery**
Individuals with candidate AMPs discovered based on species, injection method (y = acetylcholine, n = massaged), and proteomic method.

Supplemental Data 7. **DIA AMPs**
Proteomics-derived DIA candidate AMPs matching to custom AMPs database.

Supplemental Data 8. **DDA AMPs**
Proteomics-derived DDA candidate AMPs matching to custom AMPs database.

Supplemental Data 9. **Crude peptide Bd inhibition scores**
Inhibition scores of crude salamander peptides against Bd.

Supplemental Data 10. **Synthesized peptides**
Twenty peptides synthesized for challenge assays.

Supplemental Data 11. **Synthetic peptide Bd inhibition scores**
Inhibition scores of synthesized peptides against Bd.

Supplemental Data 12. **Synthetic peptide minimum inhibitory concentration (MIC) of ESPAKEE pathogens**
MIC values for synthesized peptides against ESKAPEE panel.

SUPPLEMENTARY FIGURES

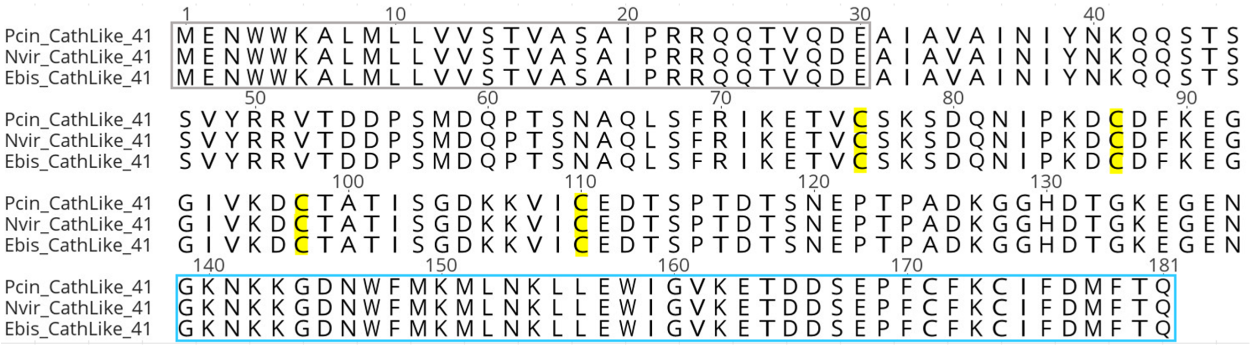

Figure S1. Cathelicidin pre-propeptide sequence diversity in the one cathelicidin peptides that was detected in all three salamander species. Grey boxed residues represent the signal peptide (pre) region. Highlighted are the four conserved cysteine residues present in the cathelin domain (pro) region. Blue boxed residues represent the predicted mature antimicrobial (peptide) region.

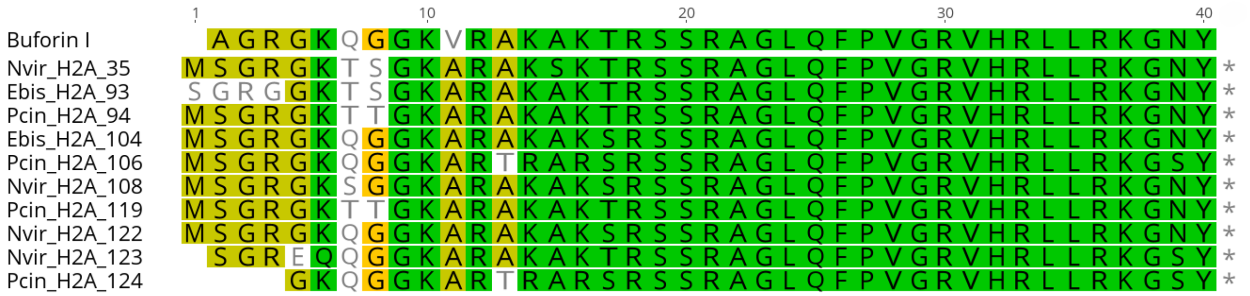

Figure S2. Histone H2A-like homology in predicted mature antimicrobial region**.** Multiple alignment of all H2A representative sequences shows interspecies homology. Green highlight represents residues conserved across all peptides. Asterisk (*) notes continuation of the sequence.

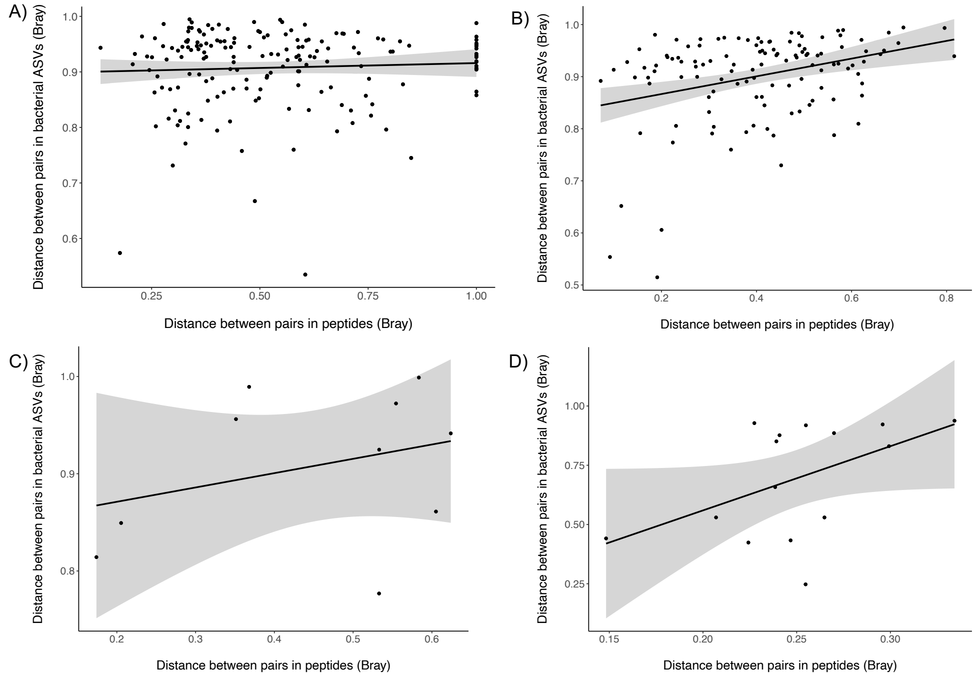

Figure S3. Scatterplots showing relationship of pairwise Bray-Curtis distances of bacterial ASV composition between pairwise Bray-Curtis distances of peptide composition in *E. bislineata* using (A) DIA and (B) DDA peptide data, and in (C) *P. cinereus* and (D) *N. viridescens* using transcriptomic peptide data.

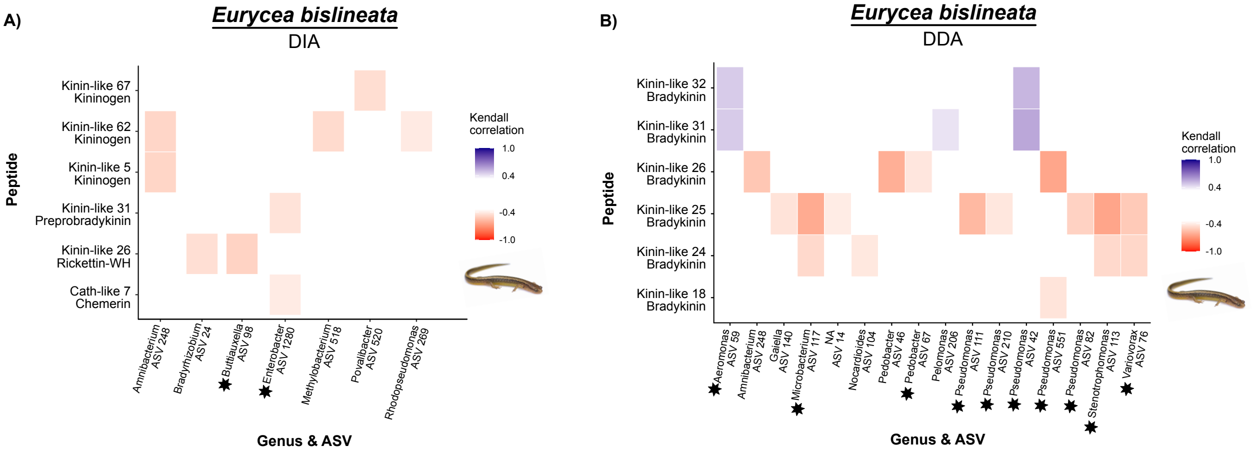

Figure S4. Heatmap of correlations of significant associations between individual candidate AMPs (unique peptide cluster that matches known AMPs database) discovered with (A) DIA and (B) DDA and individual bacterial ASVs in *E. bislineata*. Bacterial ASVs that match to known Bd-inhibitory bacteria are marked with a star.

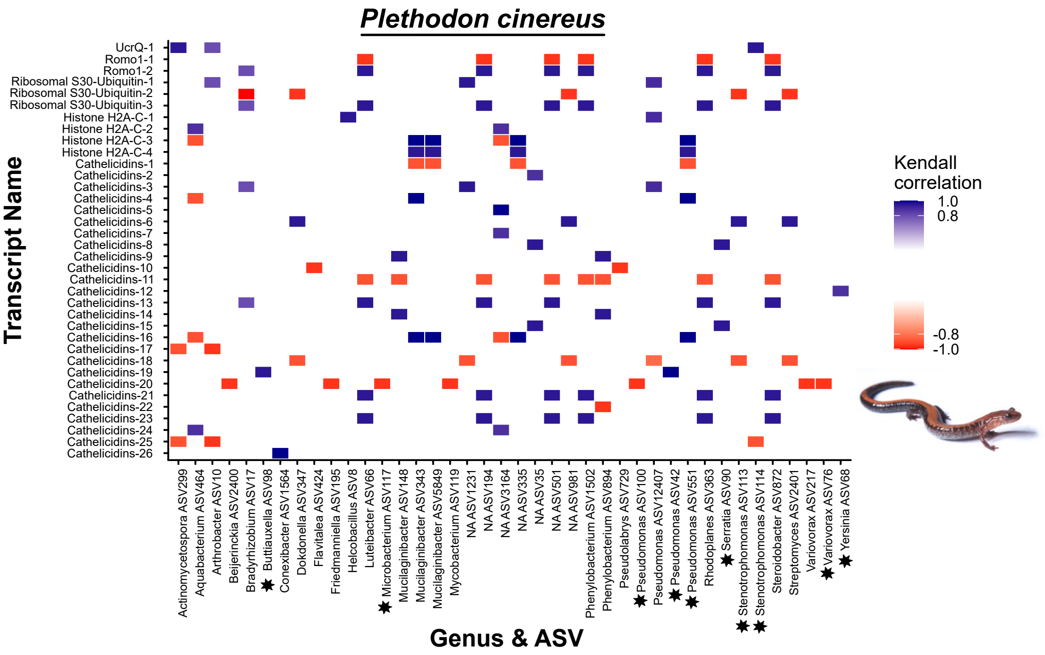

Figure S5. Heatmap of correlations of significant associations between individual candidate AMPs discovered with whole skin transcriptomics and individual bacterial ASVs in *P. cinereus*. Bacterial ASVs that match to known Bd-inhibitory bacteria are marked with a star.

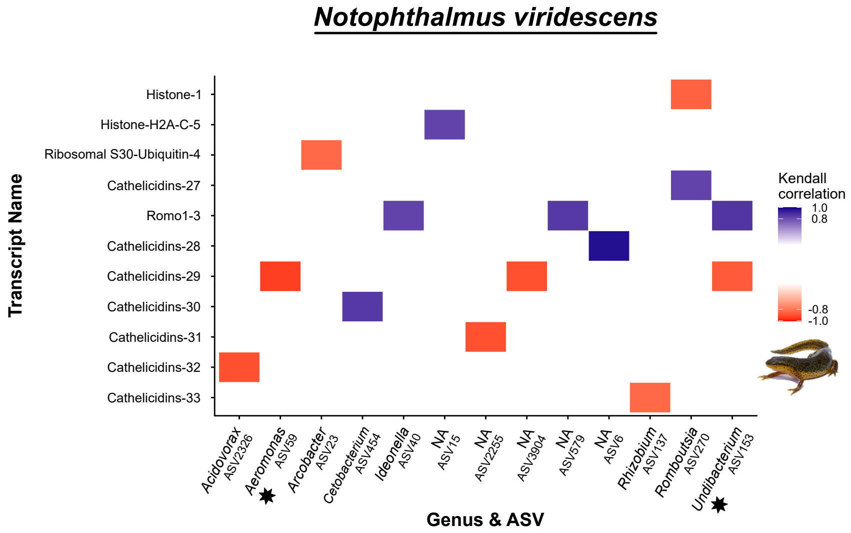

Figure S6. Heatmap of correlations of significant associations between individual candidate AMPs discovered with whole skin transcriptomics and individual bacterial ASVs in *N. viridescens*. Bacterial ASVs that match to known Bd-inhibitory bacteria are marked with a star.

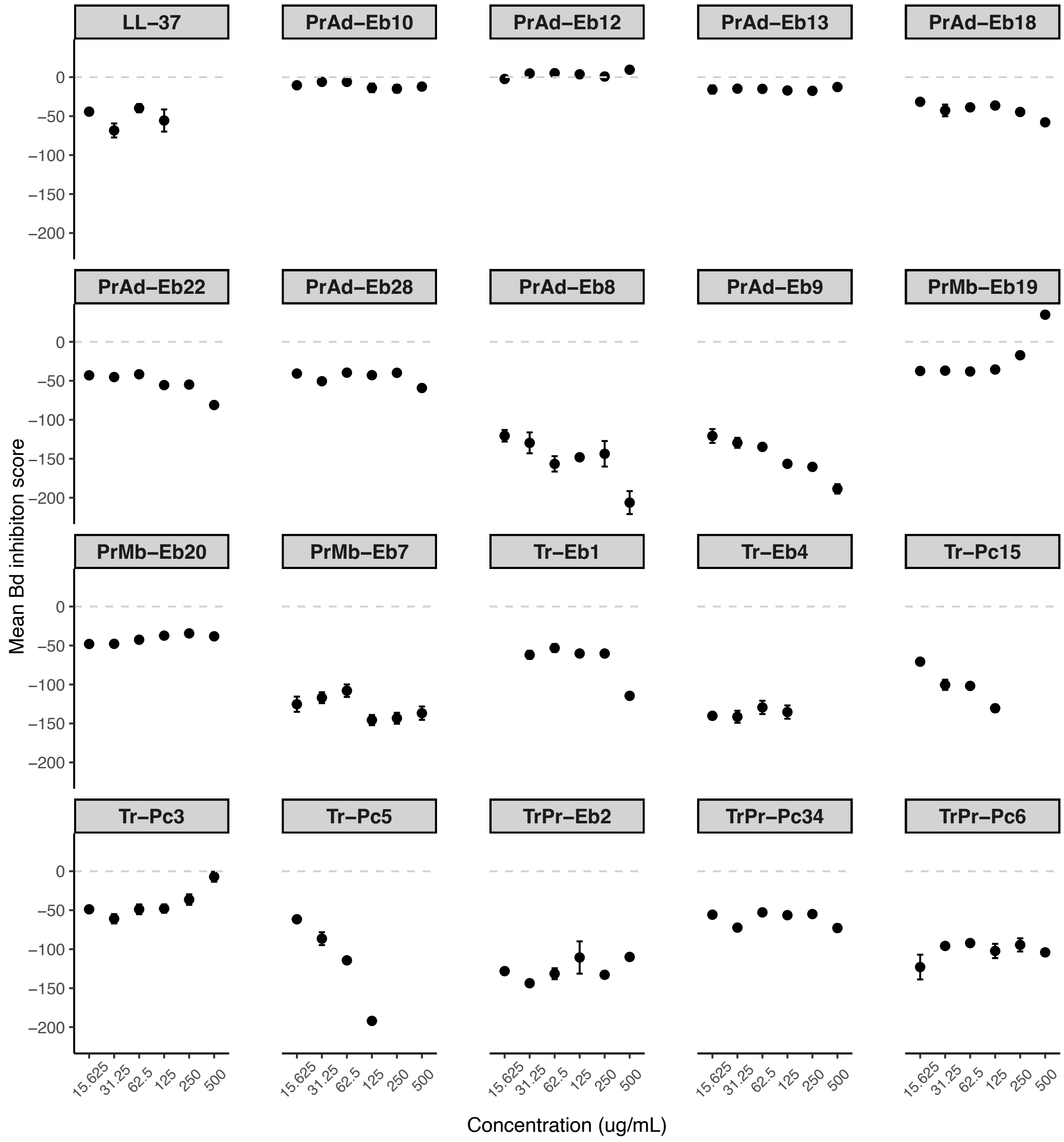

Figure S7. Mean Bd inhibition scores +/- standard error for synthesized peptides.

A)

M	L	G	W	V	R	I	L	P	L	L	T	V	A	L	T	A
T	I	P	P	L	P	Q	A	S	L	S	L	Q	D	V	V	D
V	Y	N	K	G	T	N	S	D	Y	L	Y	R	L	L	E	V
G	P	A	N	G	L	T	R	P	G	R	I	Q	F	M	V	K
E	T	E	C	L	K	S	E	M	K	N	I	E	D	C	E	F
Q	E	G	G	V	M	K	A	C	T	A	F	H	T	A	D	Q
K	T	D	D	I	V	I	N	C	D	S	P	V	L	K	R	T
R	V	R	R	A	R	P	W	N	C	Y	R	R	G	G	C	F
S	S	I	G	S	L	T	P	V	E	T	I	R	E	Q	P	Y
W	I	Q	S	P	R	L	D	S	G	T	R	A	G	L	T	P
V	E	K	F	Q	K	E	Y	G	W	I	Q	S	S	D	L	N
S	D	V	I	G	R	Y	D	S	R	L	R	P	R	D	Y	D
I	L	R	R	L	G	W	H	G	P	G	A	T	K	K		

B)

M	L	G	W	I	R	V	F	P	L	L	G	V	A	L	A	A
T	I	P	P	T	P	K	D	I	W	S	L	Q	D	V	V	D
F	Y	N	K	G	T	H	Y	D	Y	L	Y	R	L	V	D	T
A	A	S	N	V	L	A	T	P	G	R	I	Q	F	M	V	K
A	T	K	C	L	K	S	E	M	K	N	I	N	D	C	K	F
K	E	D	G	V	M	K	A	C	T	A	F	H	T	A	D	Q
K	T	D	D	I	V	I	N	C	D	S	P	V	S	K	R	T
R	V	R	R	A	R	P	W	K	C	Y	R	R	A	G	C	Y
S	I	I	G	M	F	K	K	S	P	V	K	K	L	Q	K	E
S	G	W	I	R	S	L	E	M	D	S	G	V	I	G	S	D
D	S	D	L	D	E	K	D	I	P	R	G	H	R	I	F	K
G	D	A	R	K	S	Q	E	S	S	N	P	Y	E	V	V	W
S	W	D	E	Q	D	T	K									

Figure S8. The two new Cathelicidin-like AMPs. A) Tr-Pc3 and B) Tr-Pc5 with signal peptide underlined, conserved cysteine in cathelin domain highlighted in yellow and predicted mature AMP region in the blue box.
